# Transmission dynamics of symbiotic protist communities in the termite gut: association with host adult eclosion and dispersal

**DOI:** 10.1098/rsos.231527

**Published:** 2024-05-01

**Authors:** Tatsuya Inagaki, Katsura Igai, Kazuki Takahashi, Yuichi Hongoh

**Affiliations:** ^1^ School of Life Science and Technology, Tokyo Institute of Technology, Tokyo, Japan; ^2^ Department of Entomology, Cornell University, Ithaca, NY, USA

**Keywords:** gut microbiota, mutualism, social insects, vertical transmission, caste differentiation

## Abstract

The fidelity of vertical transmission is a critical factor in maintaining mutualistic associations with microorganisms. The obligate mutualism between termites and intestinal protist communities has been maintained for over 130 million years, suggesting the faithful transmission of diverse protist species across host generations. Although a severe bottleneck can occur when alates disperse with gut protists, how protist communities are maintained during this process remains largely unknown. In this study, we examined the dynamics of intestinal protist communities during adult eclosion and alate dispersal in the termite *Reticulitermes speratus*. We found that the protist community structure in last-instar nymphs differed significantly from that in workers and persisted intact during adult eclosion, whereas all protists disappeared from the gut during moults between worker stages. The number of protists in nymphs and alates was substantially lower than in workers, whereas the proportion of protist species exhibiting low abundance in workers was higher in nymphs and alates. Using a simulation-based approach, we demonstrate that such changes in the protist community composition of nymphs and alates improve the transmission efficiency of whole protist species communities. This study thus provides novel insights into how termites have maintained mutualistic relationships with diverse gut microbiota for generations.

## 1. Introduction

The faithful transmission of microbial symbionts between host generations is a major factor in maintaining obligate mutualism. Many insects have evolved various mechanisms that ensure high fidelity of symbiont passage during vertical transmission, including symbiont invasion of oocytes before oviposition [1] or specialized structures that contain extracellular symbionts until the egg hatches [2,3]. However, studies of symbiont transmission have generally focused on relatively simple systems comprising one or two symbiont species [4–6]. Therefore, mechanistic insights into how a multi-species microbial assemblage (e.g. the gut microbiota) is maintained and transmitted between generations remain largely unexplored despite their prevalence and demonstrated functional importance [7].

Termites are a well-characterized example of obligate mutualism, as they are known for their associations with a variety of gut microorganisms that play an essential role in termite nutrition [8]. Except for the phylogenetically most apical family Termitidae, termites have symbiotic relationships with species-specific communities of protists in the hindgut [8]. This symbiosis probably originated approximately 130–150 million years ago or earlier in the common ancestor of termites and their sister lineage, the wood-feeding roach *Cryptocercus* [9–11]. These symbiotic protists are essential for host survival owing to their roles in functions such as wood digestion [12], nitrogen fixation via their endo- and/or ecto-symbiotic bacteria [12,13] and host immunity [14,15]. In many termite species, the protist community composition in the hindgut is consistent across populations [16–20], and termite-protist co-speciation has been documented [21]. These observations suggest the existence of a mechanistic basis for the faithful transmission of whole protist communities across termite generations. Recent studies have investigated the dynamics of the gut microbial community during the transmission process in termitid and non-termitid termites [22–25]. These studies have revealed that alates, winged castes that disperse from the nest, have different community compositions of protists [22,25] or bacteria [23,24] compared with workers. In the case of *Macrotermes natalensis* (Termitidae) and bacterial symbionts, alate microbiota is enriched with the termite-specific microbial lineages, leading to high transmission fidelity [23]. However, the mechanistic basis of how alates acquire such a specific composition of gut microbiota still needs to be explored in both termitid and non-termitid termites.

As intestinal protists cannot survive outside the gut of host termites, alates play a crucial role in symbiont transmission through generations in termite life history. Alates emerge from last-instar nymphs in the colony through adult eclosion and remain in the nest prior to swarming flight. To facilitate wider dispersion, their weight declines before flight, and they carry a very small number of protists [22,25,26], thus causing a significant bottleneck in protist community transmission. If the entire protist species is transmitted, this reduction in their total number will not impact transmission success. Therefore, investigating the dynamics of protist communities during this period is essential to understanding the effect of this bottleneck on the transmission success of protist species assembly. Although the dynamics of protist communities during worker development are known for some termite species, no experimental evidence of these dynamics during nymph–adult eclosion and dispersal has been reported. Workers eliminate intestinal protists before each ecdysis and regain them from surrounding nest-mates by anus-to-mouth feeding (i.e. proctodaeal trophallaxis) [27]. In contrast, preliminary observations reported in previous studies indicated that some protists remain during nymph–adult eclosion [20,27–30]. This suggests that protist community dynamics exhibit a specific pattern during nymph–adult eclosion. However, whether alates receive intestinal protists from workers before dispersal remains unknown.

**Figure 1.**
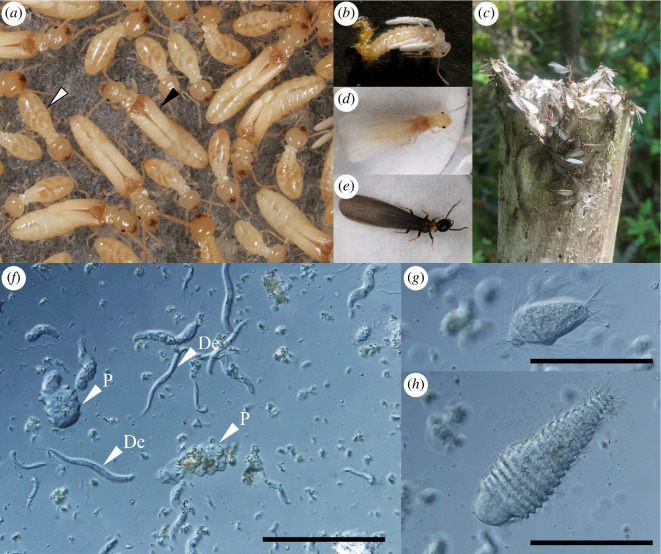
Subterranean termite, *Reticulitermes speratus*, and their symbiotic protists. (***a***) Black and white arrowheads indicate a last-instar nymph and worker, respectively. (***b–d***) The process of nymph–adult eclosion. An alate (***b***) during, (***c***) immediately after and (***d***) 7 d after eclosion. (***e***) Approximately one week after eclosion, alates congregate outside the nest and swarm. (***f–h***) Images of symbiotic protists in the gut of *R. speratus* viewed under differential interference contrast microscopy. (***f***) P: *Pyrsonympha* sp., De: *Dinenympha exilis*, (***g***) *Trichonympha agilis* and (***h***) *Teranympha mirabilis*. Scale bars (***f–h***): 100 µm.

Here, we investigated the dynamics of the protist community from nymph–adult eclosion to dispersal in the termite *Reticulitermes speratus*. In this species, newly hatched offspring differentiate into either nymph or worker castes at the third instar [31]. Both nymphs and workers grow through several moultings. Workers usually do not reproduce in the field colonies, while nymphs develop into alates and disperse to establish new colonies. Alates of this species emerge from early April to May through eclosion from last-instar nymphs [31,32] (figure 1*a*–*d*). After approximately one week, they disperse from the natal nests via simultaneous swarming [32] (figure 1*e*). *Reticulitermes speratus* is associated with a diverse community of protists comprising 10 species of the order Oxymonadida (*Pyrsonympha grandis*, *P. modesta*, *Dinenympha rugosa*, *D. exilis*, *D. porteri* type I–IV, *D. leidyi* and *D. parva*) and six species of the phylum Parabasalia (*Trichonympha agilis*, *Teranympha mirabilis, Holomastigotes* sp., *Trichomonoides* sp., *Hexamastix* sp. and *Microjoenia* sp.; figure 1*f*–*h*). Workers harbour nearly 100 000 protists in the hindgut, and the number of each protist species varies from dozens to tens of thousands [33]. Workers of most colonies harbour all of the above-mentioned protist species [17] with similar composition, suggesting faithful transmission of each protist species in *R. speratus*.

In the present study, we examined the dynamics of protist community during alate eclosion and dispersal and investigated how alates maintained whole protist species communities under significant reduction in abundances. We first compared the changes in the protist community in the gut between the worker-to-worker and nymph-to-alate moulting stages using cell counting and 18S rRNA gene amplicon sequencing. We primarily focused on counting data to investigate the degree of bottleneck effect during the transmission process. Next, we investigated whether alates obtain protists from workers in the colony prior to dispersal. We revealed that the protist community in last-instar nymphs was a primary determinant of the community in alates because alates retain protists during eclosion and disperse without receiving any protists from workers. As the community composition of nymphs differs from that of workers, we hypothesized changes in community composition in nymphs contribute to increasing the transmission efficiency of whole protist species. This hypothesis was tested by a simulation-based approach. During adult eclosion, we preliminarily observed morphological change of protist species. To evaluate the degree of morphological changes in several species of protists during adult eclosion, we designed species-specific probes, observed each protist species by fluorescent *in situ* hybridization and quantified various morphologic parameters.

## 2. Material and methods

### 2.1. Termite collection

Four colonies of *R. speratus* were collected in Japan: two in Tochigi Prefecture (designated colonies I and II) in May 2021 and two in Nagano Prefecture (colonies III and IV) in June 2021. Workers and last-instar nymphs were sampled from the nest wood of colonies I and II and used to investigate the dynamics of the protist community during worker–worker moulting and adult eclosion and dispersal (refer to §2.2 below). Colonies III and IV were used to examine whether alates obtain protists from workers in the nest (refer to §2.5 below). Last-instar nymphs were morphologically distinguished from workers and other instar nymphs by the shape of the wing pads growing from the dorsal posterolateral angles of the meso- and meta-thorax [31]. Total body weight, body weight without the gut and gut weight were measured for workers, last-instar nymphs and alates prepared in the following procedures.

### 2.2. Sample preparation

#### 2.2.1. Preparation of post-moult workers

Immediately before ecdysis, workers were sampled from two colonies. Pre-moult workers were distinguished from others by the yellowish-white coloration of the abdomen owing to the gut purge [34]. The workers were isolated in 24-well plates (Stem, Tokyo, Japan) at 25°C and observed daily for moulting. Workers were collected 2 d after moulting (moulted workers), and the absence of gut protists was confirmed by microscopic observation, as follows.

#### 2.2.2. Preparation of adult eclosion samples

**Figure 2.**
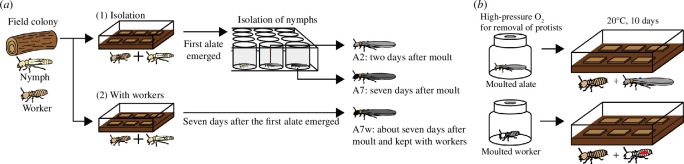
Schematic illustration of experimental procedures. (***a***) Experimental set-up for examining the dynamics of protist communities during nymph–adult eclosion and alate dispersal. Workers and nymphs collected from two colonies were kept in (1) isolation or (2) with-worker groups. In treatment 1, nymphs were isolated after the first alate emerged in the plastic box and observed daily. Two days (**A2**) and seven days (**A7**) after adult eclosion, alates were collected. In treatment 2, alates were collected 7 d after the first alate emerged in the box (A7w). After collection, individuals were dissected, and the protists were counted under a microscope. (***b***) Set-up for measuring the number of protists obtained from workers after moulting. Alates that moulted within 2 d were treated with high-pressure O_2_ (100% O_2_, 0.3 MPa for 1 h and 0 MPa for 23 h) to eliminate all protists. The alates were then kept with workers in a plastic box for 10 d at 20°C. Workers were used as a control. Moulted workers were marked with red ink to distinguish them from surrounding workers.

Workers and last-instar nymphs from colonies I and II were subjected to one of two treatments: (1) isolation or (2) being kept with workers (figure 2*a*). The sex of nymphs and alates was determined based on the morphology of the seventh and eighth sternites according to previous reports [35,36]. In treatment 1, nymphs and workers were kept together at 20°C in several plastic cases (221 × 141 × 37 mm³, Mizuho-kasei, Nagoya, Japan). The cases contained brown rotten wood powder mix (brown rotten wood powder:cellulose = 1:1, [37]) and slices of Douglas fir lumber. We observed the cases daily to assess differentiation from nymphs to alates. After the first alate emerged, we extracted all of the remaining nymphs and isolated them in 24-well plates. Filter paper (Whatman No. 2, 15 mm radius, Cytiva, Marlborough, MA, USA) and 50 μl of double-distilled water were added to each well. The last-instar nymphs in the wells were observed daily, and the day of alate differentiation was recorded. Alates were extracted on the day of differentiation (A0) and 2 d (A2) after differentiation. Remaining alates were placed in the wells containing brown rotten wood powder mix and then extracted 7 d after differentiation (A7).

In treatment 2, a total of 20 male and 20 female nymphs were kept at 20°C with 500 workers in a plastic case (100 × 100 × 29 mm³) with brown rotten wood powder mix and slices of Douglas fir lumber. Nymphs were observed daily from the bottom of the case. One week later, we observed the first alate differentiation, the alates were extracted as A7w. At that time, most of the alates had moved to the top of the case and were about to swarm.

### 2.3. Cell counting and observation of protists

The hindgut was extracted from each termite by gently pulling the tip of the abdomen using forceps. The gut contents were homogenized in Trager’s solution U [38]. The volume of solution was adjusted for each caste/group (worker: 150 μl, last-instar nymph, A2, A7, A7w: 30 µl). For all castes/groups, 20 μl of the resulting solution was loaded into a C-chip haemocytometer (NanoEntek, Seoul, Korea), and the protist cells were counted for each oxymonad and parabasalid species under a differential interference contrast microscope (IX81; Olympus, Tokyo, Japan). *Reticulitermes speratus* workers harbour up to 16 morphologically distinguishable species of protists in the hindgut [39–41]. We categorized protists as follows based on the description of their morphologies in previous studies [39–41] for convenience, as some protist species can be difficult to discriminate using live specimens: *Pyrsonympha* spp. (*P. grandis* and *P. modesta*), *Dinenympha exilis*, *D. porteri* type III and IV, *Dinenympha* spp.1 (*D. rugosa* and *D. porteri* type I and II), *Dinenympha* spp.2 (*D. leidyi* and *D. parva*), *Tr. agilis*, *Te. mirabilis*, *Holomastigotes* sp. and small protists (*Trichomonas* sp., *Hexamastix* sp., and *Microjoenia* sp.). The large protists *Tr. agilis* and *Te. mirabilis* were counted in an area comprising 16 squares (3.2 μl) of the haemocytometer. Other protists were enumerated by counting within four squares at the corners (0.8 μl). The population size of each protist group was estimated, and these populations were summed to determine the total number of protists in each termite. The estimates were rounded down to the nearest integer. After determining the number of protists, the weight of the remaining body parts was determined, and the weight of the gut of each individual was estimated.

### 2.4. 18S rRNA gene amplicon sequencing analysis of protist community

18S rRNA gene amplicon sequencing analysis of workers, nymphs and A7 (isolated, 7 d post-eclosion alates) was carried out using oxymonad- and parabasalid-specific primer sets, respectively. Whole-gut DNA was extracted from individual samples using Nucleo-Spin Tissue XS extraction kits (Macherey-Nagel, Düren, Germany) according to the manufacturer’s instructions, with some modifications [22]. Before the lysis step, a sterile stainless-steel bead (5 mm, Qiagen, Hilden, Germany) was placed in each tube, and the sample tubes were attached to a Vortex Adapter (24 tubes, Qiagen) and homogenized using a vortex mixer (Vortex-GENIE 2, Scientific Industries, Bohemia, NY, USA) at maximum speed for 10 min. The V3–V4 regions of 18S rRNA genes were polymerase chain reaction (PCR)-amplified using the oxymonad-specific primers [41], with some modifications (RsOx_Sc432: 5′-GCGCAAATTACCCACTGGCA-3′; RsOx_Sc789_2Y: 5′-TTCAGCYGCGARACGCCYTG-3′), and the parabasalid-specific primers [22] (Par_18 S-F: 5′-GCAGCAGGCGYGAAAC-3′; Par_18 S-R: 5′-CCTACTCTCGCYCTTGATCG-3′). The PCR mixture contained 1 μl of whole-gut DNA, 1 × HF buffer, 0.2 mM dNTPs, 0.5 μM primer set, and 0.4 U of Phusion high-fidelity DNA polymerase (New England Biolabs, Ipswich, MA, USA). The thermal conditions were as follows: initial denaturation for 30 s at 98°C, 25 cycles of denaturation at 98°C for 10 s, annealing at 55°C for 15 s, extension at 72°C for 30 s and final extension at 72°C for 5 min. The PCR products were purified using Agencourt AMPure XP (Beckman Coulter, Brea, CA, USA), and DNA concentration was determined using the Qubit dsDNA HS assay kit (Thermo Fisher Scientific, Waltham, MA, USA). The purified PCR products (approx. 0.5 ng) were subjected to PCR amplification to attach Nextera XT indices (Illumina, San Diego, CA, USA) under the following conditions: 98°C for 30 s; eight cycles of 98°C for 10 s, 55°C for 30 s, 72°C for 45 s and 72°C for 7 min. Sequencing was conducted using an Illumina MiSeq platform with the MiSeq Reagent kit v3 (600 cycles).

For demultiplexing of paired-end reads, we used the clsplitseq command in the Claident package (https://www.claident.org/) [42] with a minimal quality value (Phred score) of 30 (--minqual tag = 30). Subsequently, the demultiplexed paired-end reads were quality filtered and trimmed using the dada2 v. 1.6 program package [43] with the following parameter settings: truncLen = c(280, 250), maxEE = c(2, 5) and truncQ = 2. To compare the diversity of protists among all samples under similar sequencing conditions, the quality-filtered reads of each sample were rarefied to 10 000 read pair sets. The rarefied reads were combined (i.e. 470 000 and 450 000 read pairs in total for Oxymonadida and Parabasalia, respectively), followed by denoising, merging of paired-end reads, identification of chimeras and sorting into single-nucleotide-level amplicon sequence variants (ASVs) using dada2. ASVs with a relative abundance of less than 0.1% of the total reads in a given sample and those detected in only one sample were excluded based on previous control experiments [41]. ASVs were subsequently grouped into operational taxonomic units (OTUs) based on 97% nucleotide similarity using the DECIPHER package in R [44]. Samples that did not yield a sufficient amount of DNA at each stage of analysis were excluded. Ultimately, three–five replicates were used for workers and each sex of nymphs and A7.

For the taxonomic assignment of oxymonad and parabasalid OTUs, OTU sequences were aligned with the corresponding region of the near-full-length 18S rRNA sequences retrieved from NCBI nr Database using MAFFT [45] with the ‘auto’ option; ambiguously aligned sites were trimmed using TrimAl [46] with the ‘nogaps’ option. Maximum-likelihood trees were constructed using IQ-TREE v. re-1.6.12 [47] with the TIM3 + F + I + G4 and TIM2e + G4 models for oxymonad and parabasalid trees, respectively, with ultrafast bootstrap analysis [48] with 1000 resamplings.

### 2.5. Removal of protists using oxygen gas

To examine if alates and workers regain protists from workers after their moult, we removed gut protists by treating moulted alates and moulted workers with oxygen gas, kept them with non-treated workers, and then compared the number of protists with those of treated alates and workers (figure 2*b*). To obtain moulted alates, last-instar nymphs were placed in a Petri dish with moist unwoven cloth and maintained at 20°C until they became alates by eclosion. Alates within 2 d after moult (16 males and 20 females) were transferred into a rubber-stoppered gas chromatography vial (net capacity 30 ml, SVG-30, Nichidenrika-Glass, Kobe, Japan) for treatment with oxygen (figure 2*b*). The inner bottom of each glass vial was lined with moist unwoven cloth. An oxygen spray can (Nitto Kagaku, Nagoya, Japan) was connected to the vial via an injection needle, and an oxygen concentration meter (Ichinen Jikco, Nagoya, Japan) was also connected to the glass vial via another needle with the air being allowed to flow out. Oxygen was loaded into the vials until the concentration reached approximately 100%, after which the oxygen concentration meter was disconnected and a pressure gauge (APG-1, Fujiwara Sangyo, Hyogo, Japan) connected. Oxygen was loaded into the vial again until the pressure reached 0.3 MPa. The alates were kept in the presence of high-pressure oxygen for 1 h, after which the vials were depressurized by insertion of an injection needle, and the alates were kept in the vials for another 23 h. Four males and four females were used to confirm the successful elimination of protists, and 12 male and 16 female alates were transferred and kept with 500 workers in plastic cases containing brown rotten wood powder mixed with slices of Douglas fir lumber at 20°C. After 10 d, surviving alates (four males and seven females in each colony) were collected, and the number of protists was determined as described in §2.3. To obtain moulted workers, pre-moult workers were collected as described above. Forty-four workers within 0–3 d after moult were treated under high oxygen conditions in the same manner as the alates. After oxygen treatment, six workers per colony were examined to confirm the absence of protists in the gut. The dorsal side of the abdomen of each post-moult worker was marked with red oil-based paint (Mitsubishi pencil, Tokyo, Japan) to distinguish it from other workers in the plastic case. The painted workers were kept with 500 workers in the plastic cases containing brown rotten wood powder mixed with slices of Douglas fir lumber at 20°C. After 10 d, 22 post-moult workers were collected, and the number of protists was determined as described above.

### 2.6. Simulation of vertical transmission of the protist community

A computer simulation was conducted to examine the potential impact of changes in protist community composition in nymphs on the transmission efficiency of the entire protist community. We first deliberately assumed an unrealistic situation, where protists were transmitted from workers to alates. Next, we assumed a realistic situation, where protists were maintained through nymphs to alates. Then, the minimum number of protists required for alates to transmit all protists species was calculated in two scenarios. To clarify whether the protist abundance of alates is sufficient to achieve high transmission efficiency, we also compared the results with empirical data.

We performed the sampling with replacement from a hypothetical protist community in workers or nymphs by selecting 500 to 10 000 protist cells (in increments of 500). The community compositions in the hypothetical workers and nymphs were determined as the average of empirical data from colonies I and II (electronic supplementary material, table S1). Sampled protists were then introduced into protist-free hypothetical alates. Once the total number of protists reached its maximum number in hypothetical alates, each alate was assessed for the presence of each protist species. We calculated the transmission efficiency, which is defined by the proportion of alates that have all protist species after 5000 iterations. The R code used for these simulations is available at https://github.com/TatsuyaInagaki2/Data_alate_protist_community.

### 2.7. Morphological observation of protists during adult eclosion

We designed oligonucleotide probes to specifically target 18S rRNA specific to *D. exilis* and *D. leidyi* using the probe-designing function in ARB [49]. The probe sequences were 5ʹ-ACGCACAGGGAAAACGAA-3ʹ for *D. exilis* and 5ʹ-GAATGAAGCATCGGCACG-3ʹ for *D. leidyi*. 6-Carboxyfluorescein (6FAM) and Texas red were attached to the 5ʹ ends of the probes for *D. exilis* and *D. leidyi*, respectively. The gut contents of eight individual workers and four males and four female nymphs, A0, A2 and A7w from each colony were examined. Fixation and hybridization were performed as described previously [50,51], with slight modifications. Specimens were subjected to prehybridization in buffer (0.9 M NaCl, 0.1 M Tris-HCl) at 48°C for 15 min followed by hybridization with the probes at 48°C for 3 h. Samples were observed using an epifluorescence microscope (BX51, Olympus, Tokyo, Japan). Probe specificity was confirmed using specimens from gut samples of workers. Protist images were processed using Adobe Photoshop CC. The edges of cells were trimmed using the Quick Selection Tool function, and the area (µm^2^) and circularity (4П [area/perimeter^2^]) were determined, with a circularity value of 1.0 indicating a perfect circle. We processed 25 cells of each protist species from each caste/group (worker, nymph, A2, A7 and A7w) of the two colonies.

### 2.8. Statistical analysis

The total number of protists, fresh body weight, body weight without gut and gut weight in each caste/group (worker, nymph, A2, A7 and A7w) were compared using linear mixed models (LMMs) and likelihood ratio tests (LRTs). In the LMMs, caste/group was included as an explanatory variable and colony as a random factor. To elucidate the effect of nymph and alate sex, we also ran LMMs including sex, caste/group and their interaction as explanatory variables and colony as a random factor.

To examine the effects of colony and caste/group on protist communities, we conducted permutational analysis of variance (PERMANOVA) on Bray–Curtis dissimilarity indices calculated from cell count data. Colony, caste/group and their interaction effect were included as explanatory variables. To elucidate the effect of sex in nymphs and alates (A2, A7 and A7w), we also ran PERMANOVA including sex, caste/group and colony as explanatory variables. The effects of colony, caste/group and their interaction on the proportions of *Tr. agilis* and *Te. mirabilis* in cell count data were tested using a generalized linear model (GLM) with quasi-binomial error distribution, as the data were over-dispersed.

For read counts of oxymonad and parabasalid OTUs derived from amplicon sequencing, we performed non-metric multi-dimensional scaling (NMDS) and PERMANOVA on Euclidean distances calculated from centred log-ratio-transformed data owing to the data compositionality [52,53]. The effects of colony, caste (worker, nymph and A7) and their interaction were tested. To elucidate the effect of nymph and A7 sex, we also ran PERMANOVA including sex, caste and colony as explanatory variables.

To compare the area and circularity of two oxymonad protist species, we conducted pairwise Wilcoxon tests among castes/groups (worker, nymph, A0, A2 and A7w) in each colony.

LMMs were conducted using the *lme4* package [54], and PERMANOVAs were performed using the *vegan* package [55] in R.

## 3. Results

### 3.1. Persistence of protist communities during adult eclosion from nymphs to alates

**Figure 3.**
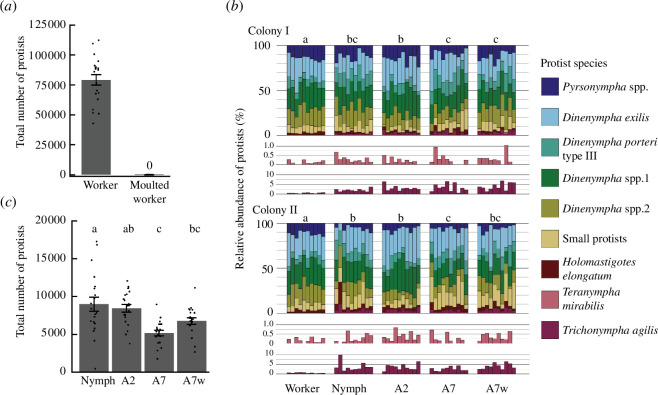
Dynamics of intestinal protist communities during alate differentiation and dispersal. Changes in the number of protist cells during (***a***) worker moulting and (*c*) nymph–adult eclosion and alate dispersal are shown. Different characters denote significant differences. LMM, colony was included as a random factor, Tukey’s HSD, *p* < 0.05. In (***a***) and (*c*), error bars denote the standard error, and points are individual measurements. (*b*) Comparison of protist communities derived from cell counting among castes/groups. Different characters indicate significant differences between protist communities. PERMANOVAs were conducted between castes/groups in each colony (Bray–Curtis dissimilarity, 9999 permutations, Bonferroni correction, corrected *α* = 0.005). The relative abundance of *Te. mirabilis* and *Tr. agilis* in each sample is additionally shown at the bottom.

Workers from the field colonies harboured a number of protists in their gut, whereas none retained any protist cells immediately after moulting (workers: 79248.6 ± 4432.9, moulted workers: 0 ± 0, mean ± s.e., figure 3*a*). In contrast, the number of protists did not change significantly during nymph–adult eclosion (nymphs: 8992.1 ± 933.2, A2: 8457.0 ± 503.8, figure 3*b*). Seven days after eclosion, the number of protists significantly decreased in A7 (5193.5 ± 370.1, figure 3*b*). The decrease in protists in A7w was smaller (6770.2 ± 428.5, figure 3*b*), but the number of protist cells was not significantly different from that of A7. Among the nymphs and alates examined, the sex of individuals and interaction between sexes and castes/groups had no significant effect on the total number of protists (LMM, LRT, caste/group: $\chi_{3}^{2}$ = 23.73, *p* < 0.001; sex: $\chi_{1}^{2}$ = 0.77, *p* = 0.38; caste/group × sex: $\chi_{3}^{2}$ = 0.81, *p* = 0.85).

The effects of caste/group and colony on the protist community composition based on cell counts were significant, whereas the interaction effect was not (PERMANOVA, caste/group: *F_4_* = 56.73, *p* < 0.001; colony: *F_1_* = 5.99, *p* = 0.004; caste/group × colony: *F_4_* = 1.22, *p* = 0.274). In both colonies I and II, the protist community composition did not change before or after adult eclosion and was not affected by worker presence (figure 3*c*). PERMANOVA of nymphs and alates with colony, caste/group and sex as explanatory variables showed no significant effect of individual sex on protist community composition (*F_1_* = 1.39, *p* = 0.22). When focusing on *Te. mirabilis* and *Tr. agilis*, which showed the lowest and second lowest abundance in workers, respectively, we found that proportions of both species increased in nymphs and alates (figure 3*c* and electronic supplementary material, figure S1). The proportion increased approximately sixfold in *Tr. agilis* (percentage proportion in workers: 0.48 ± 0.05, nymphs: 2.93 ± 0.44, A2: 3.30 ± 0.32, A7: 2.93 ± 0.36, A7w: 3.98 ± 0.37, mean ± s.e., electronic supplementary material, figure S1) and twofold in *Te. mirabilis* (percentage proportion in workers: 0.14 ± 0.03, nymphs: 0.27 ± 0.04, A2: 0.29 ± 0.05, A7: 0.24 ± 0.06, A7w: 0.31 ± 0.06, electronic supplementary material, figure S1). There were no significant differences between colonies in terms of the proportions of *Tr. agilis* (GLM, LRT, colony: $\chi_{1}^{2}$ = 1.2, *p* = 0.27; caste/group: $\chi_{4}^{2}$ = 422.4, *p* < 0.001; colony × caste/group: $\chi_{4}^{2}$ = 5.8, *p* = 0.21) and *Te. mirabilis* (GLM, LRT, colony: $\chi_{1}^{2}$ = 2.4, *p* = 0.122; caste/group: $\chi_{4}^{2}$ = 18.8, *p* < 0.001; colony × caste/group: $\chi_{4}^{2}$ = 1.5, *p* = 0.83).

In the amplicon sequencing analyses of oxymonad protists, we assigned 20 OTUs to nine species-level taxa belonging to two genera (*Pyrsonympha* and *Dinenympha*; electronic supplementary material, figure S2). For parabasalid protists, we assigned 10 OTUs to eight species-level taxa (electronic supplementary material, figure S3). The oxymonad community differed significantly among castes (workers, nymphs and A7) and between colonies, whereas the interaction effect of colony and caste was not significant (PERMANOVA, caste: *F_2_* = 2.9, *p* < 0.001; colony: *F_1_* = 5.8, *p* < 0.001; caste × colony: *F_2_* = 1.4, *p* = 0.08, electronic supplementary material, figure S4). We also detected significant differences in the parabasalid community among castes and between colonies (caste: *F_2_* = 5.6, *p* < 0.001; colony: *F_1_* = 2.7, *p* = 0.01; caste × colony: *F_2_* = 1.4, *p* = 0.15, electronic supplementary material, figure S4), but the difference between colonies was less clear compared with the oxymonad community based on NMDS (electronic supplementary material, figure S4). PERMANOVA of nymphs and alates with colony, caste and sex as explanatory variables showed no significant effect of sex on either oxymonad (*F_1_* = 1.11, *p* = 0.33) or parabasalid (*F_1_* = 1.1, *p* = 0.33) community composition. All oxymonad protists were detected in workers, whereas five species were not detected in several samples of nymphs and alates (electronic supplementary material, figure S4 and table S2). Among nymphs, the detection ratios of *D. leidyi*, *D. porteri* type I and *D. parva* were 0.947, 0.947 and 0.579, respectively, and among alates, the ratios of *P. modesta*, *D. rugosa*, *D. porteri* type I and *D. parva* were 0.772, 0.944, 0.778 and 0.722, respectively (electronic supplementary material, table S2). Among parabasalid protists, *Tr. agilis* and *Te. mirabilis* were detected in all nymphs and alates and most workers, whereas the detection rates of *Holomastigotes* sp. or *Microjoenia* sp. were lower in alates and nymphs than in workers. The detection rates of unclassified Trichomonadidae 1 and 2 were relatively low in all castes compared with other parabasalids (electronic supplementary material, table S3).

Total body weight, body weight without the gut and gut weight differed significantly among workers, nymphs, A2, A7 and A7w (LMM, LRT, fresh weight: $\chi_{5}^{2}$ = 1454.7, *p* < 0.001; body weight: $\chi_{5}^{2}$ = 1562.5, *p* < 0.001; gut weight: $\chi_{5}^{2}$ = 218.9, *p* < 0.001, electronic supplementary material, figure S5). Nymphs and alates showed higher fresh weight and body weight than workers, whereas the gut weight of workers was similar in A2 but heavier than that of nymphs, A7 and A7w (electronic supplementary material, figure S5). Among A2 and A7w nymphs, females showed higher fresh weight and body weight than males, whereas the gut weight did not differ significantly between sexes (electronic supplementary material, figure S5).

### 3.2. No transmission of protists from workers to alates

After oxygenation treatment to eliminate protists in the gut and keeping with non-treated workers for 10 d, moulted alates did not regain protists at all, while moulted workers regained protists in their gut (alate: 0 ± 0, worker: 126478.8 ± 5606.3, mean ± s.e.). The entire gut fluid suspensions of alates were examined and no protist cell was observed from any samples. This suggests that alates did not obtain protists from workers via proctodaeal trophallaxis after their eclosion.

### 3.3. Changes in the community composition of nymphs contribute to transmission efficiency

**Figure 4.**
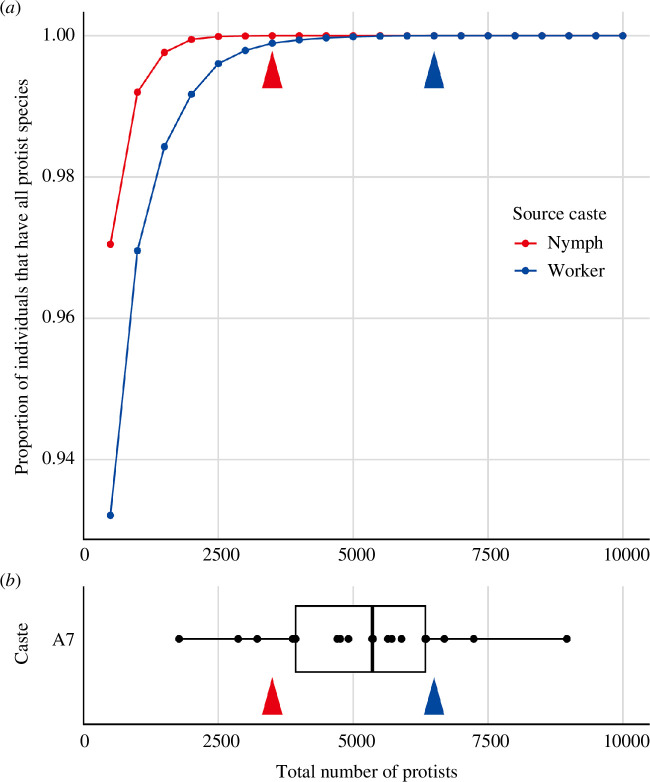
Changes in the protist community composition of nymphs improve transmission efficiency. (***a***) Simulation of the protist transmission from nymphs or workers to alates. Higher transmission efficiency (i.e. ratio of alates that have all protist species/groups) can be achieved with fewer numbers when protists were transmitted from nymphs than when transmitted from workers. Minimum number of protists required was 3500 (red arrowhead) and 6000 (blue arrowhead) when transmitted from nymphs or workers, respectively. (***b***) Such difference is critical for alates since most of them had more than 3500 but less than 6000 cells. Boxplot shows the total number of protists in alates (A7: alate isolated 7 d after eclosion). Points are individual measurements, and boxplot shows the range, median and quartiles for each treatment.

Through a computer simulation, we investigated the advantage to maintain protist communities from nymphs to alates. Figure 4*a* shows the transmission efficiency (i.e. the proportion of hypothetical alates with all protist species) based on two scenarios: transmission of protist communities from workers or nymphs. The minimum number of protists cells required to ensure that all protist species would be transmitted (i.e. transmission efficiency of 100%) differed between the two scenarios: 3500 and 6500 protist cells were required to be transmitted from nymphs and workers, respectively. This difference was attributed to the observation that *Te. mirabilis*, which exhibited the lowest proportion of the protist community, comprised a greater proportion of the community in the gut of nymphs than in that of workers. A comparison with the empirical data (figure 4*b*) showed that most A7 alates (17/20) harboured greater than 3500 protist cells, but very few individuals (3/20) harboured greater than 6500 cells. This result suggests that in the case of random protist transmission from workers, some alates would lack one or more protist species. Therefore, nymphs should be the source of the protist community of alates owing to higher transmission efficiency.

### 3.4. Protist species-specific changes in cell morphology during alate ecdysis

Our preliminary observation showed that the morphology of *D. leidyi* changes significantly during adult eclosion, whereas that of *D. exilis* does not. To evaluate the degree of morphological changes in these two species during adult eclosion, we designed species-specific probes, observed each protist species using FISH and quantified various morphologic parameters (electronic supplementary material, figure S6). With the exception of the area of *D. exilis* in colony IV, the area and circularity of both *D. leidyi* and *D. exilis* changed significantly during adult eclosion (electronic supplementary material, figure S7). In the gut of workers, the size of *D. leidyi* cells varied widely, and many cells were slightly twisted (electronic supplementary material, figures S6 and S7). During nymph–adult eclosion, the cells became much smaller and more circular (electronic supplementary material, figures S6 and S7), suggesting that they were strongly folded during this period. After eclosion, the cells became larger, and their shape returned to a form more like that of workers (electronic supplementary material, figures S6 and S7). In contrast, few changes were observed for *D. exilis* (electronic supplementary material, figures S6 and S7). These cells almost always maintained an elongated shape, even though the cells were rounded slightly in the gut of nymphs (electronic supplementary material, figures S6 and S7). No apparent changes in the morphology of the parabasalid species *Tr. agilis* and *Te. mirabilis* were observed during termite adult eclosion.

## 4. Discussion

We characterized the caste-specific dynamics of the protist community during development of the termite *R. speratus*. In worker–worker moults, no protists were retained during moulting (figure 3*a*), and the workers obtained protists from nest-mates. In contrast, in nymph–adult eclosion, individual termites retained almost all protists (figure 3*b*,*c*) and never obtained them from workers. Therefore, the protist community of nymphs is the primary determinant of the community of alates, and maintenance of the protist community during eclosion is essential for successful transmission. Our results also showed that the number of protists in nymphs and alates was dramatically lower than that of workers (figure 3*a*,*b*), probably because alates must reduce their weight for further dispersal (electronic supplementary material, figure S5).

In nymphs and alates, the proportions of *Tr. agilis* and *Te. mirabilis*, which exhibited the lowest proportions in workers, increased markedly (figure 3*c* and electronic supplementary material, figure S1). Our simulation-based analysis revealed that such changes in the protist community in nymphs enhance the transmission efficiency of the complete assembly of protist species. This is because higher transmission efficiency (i.e. ratio of alates that have all protist species/groups) can be achieved with fewer numbers when transmitted from nymphs than when transmitted from workers (figure 4*a*). When we deliberately assumed the unrealistic scenario where protists were transmitted from workers, alates would need to carry more protists to transmit all protists than they actually had (figure 4*b*). Therefore, changes in the composition of the protist community in nymphs and maintenance of the composition until alate dispersal play important roles in ensuring the successful transmission of all protist species. Besides, both above-mentioned protist species beneficially affect the host: *Tr. agilis* and *Te. mirabilis* cells are large (figure 1*g*,*h*) and can, therefore, digest large wood particles using cellulase [56,57]. The symbiotic bacteria associated with both protist species contribute to the termite-gut ecosystem by supplementing various nitrogenous compounds and by preventing the accumulation of molecular hydrogen, which otherwise would suppress the fermentation of cellulose [13,58,59]. Thus, compositional changes of protist community in the nymphs may also contribute to maintaining the function of termite gut microbiota in newly founded colonies.

In contrast, our amplicon sequencing analysis revealed that several protists other than *Tr. agilis* and *Te. mirabilis* were not detected from some nymphs and alates (electronic supplementary material, tables S2 and S3), suggesting that not all protist species are completely maintained throughout the transmission process. This result was consistent with previous studies examining *Reticulitermes grassei* [22] and *Coptotermes* sp. [25]. Velenovsky et al. [25] suggested that biparental transmission contributes significantly to the high rates of occurrence of protist species in field colonies of *Coptotermes* sp. In termites, alates usually form monogamous pairs to establish a new colony, and both founders engage in foraging, reproduction and caring for larvae via anus-to-mouth feeding [60]. Previous research showed that the number of protists in both male and female founders increases dramatically after colony founding [61–63]. As no significant sex-related differences in the number or community composition of protists or the weight of the gut in nymphs and alates were observed in the present study, the contribution of both sexes as carriers of the protist community would be essentially equal. This suggests that the biparental transmission may increase the likelihood of protist species with small numbers to be transmitted to the next generation rather than that alates carry protist species specific to each sex. However, we investigated a limited number of samples, since the present study was based on manipulative experiments requiring multiple steps (figure 2) and alates only appear in a very limited period annually. Therefore, it is possible that sex differences in protist community of alates are colony-dependent as shown in *R. grassei* [22]. Further investigation on alate protist communities is needed to understand the role of alate sex on protist transmission.

Our results showed that the maintenance of protist communities during eclosion is essential for successful transmission. As the conditions in the hindgut can change dramatically during adult eclosion owing to the shedding of the epithelium of the hindgut wall [28], protists may have to deal with unusual environmental changes during eclosion. We observed synchronized morphological changes in certain protist species during the adult eclosion of termites. The size of *D. leidyi* cells decreased, and the cells appeared to be strongly folded just after moulting (electronic supplementary material, figures S6 and S7). The cells returned to their normal shape in the alates 7 d after eclosion (electronic supplementary material, figures S6 and S7). Such changes during host eclosion have been reported in various termite species [20,30,64]. For instance, cells of the oxymonad protist *Streblomastix strix* harboured by termites of the genus *Zootermopsis* usually exhibit an elongated morphology, but they exhibit a rounded shape during adult eclosion [30]. These observations suggest that morphological changes in some protist species enable them to withstand the environmental changes in the hindgut associated with adult eclosion. In contrast, the morphology of *D. exilis* exhibited only slight changes during adult eclosion (electronic supplementary material, figures S6 and S7). Strategies for overcoming environmental changes may differ between protist species. Although why and how such species-specific morphological changes are induced during adult eclosion remain to be investigated, future studies focusing on physiological changes in the gut environment and protists might address these questions.

Protist communities in the termite gut are maintained within colonies and between generations. The present study suggests that the assembly of the alate protist microbiota is regulated to maximize chances of vertical transmission for whole protist species. This should help maintain the metabolic function of gut microbiota in termites, probably because the role of each protist differs, as shown in the termite *Coptotermes formosanus* [65]. Although recent studies have investigated the dynamics of gut microbiota in termite life history [8–10,61], the relationship between termites and each microbe is still largely unknown. Future studies are needed to understand the mechanistic basis of maintaining gut microbiota in each caste by investigating the food, trophallactic behaviour and physiology. Moreover, investigating the fitness effect of each microbe on the host is also essential to understanding the adaptive significance of termites to maintain diverse microbes. Artificial manipulation of microbial communities, such as the selective elimination of specific microbes and investigation of host fitness, will also enhance our understanding of the relationship between termites and each microbial species. These approaches will contribute to a deeper understanding of the ecology and evolution of their long-standing symbiotic relationships.

## Data Availability

Data and relevant code for this research work are stored in GitHub: https://github.com/TatsuyaInagaki2/Data_alate_protist_community and have been archived within the Zenodo repository [66]. Additional information is provided in the electronic supplementary material [67]. The demultiplexed MiSeq datasets have been deposited in the DDBJ Sequence Read Archive under the BioProject PRJDB15577 with BioSample accession nos. SAMD00589838–SAMD00589842, SAMD00598142–SAMD00598186.
